# Conserved motif VIII of murine DNA methyltransferase Dnmt3a is essential for methylation activity

**DOI:** 10.1186/s12858-016-0064-y

**Published:** 2016-03-22

**Authors:** Olga V. Lukashevich, Natalia A. Cherepanova, Renata Z. Jurkovska, Albert Jeltsch, Elizaveta S. Gromova

**Affiliations:** Department of Chemistry, Moscow State University, 119991 Moscow, Russia; Institute of Biochemistry, Faculty of Chemistry, University Stuttgart, Pfaffenwaldring 55, 70569 Stuttgart, Germany; BioMedX Innovation Center, Im Neuenheimer Feld 583, 69120 Heidelberg, Germany

**Keywords:** Dnmt3a DNA methyltransferase, DNA methylation, DNA binding, Dnmt3a mutants, Catalytic domain

## Abstract

**Background:**

Dnmt3a is a DNA methyltransferase that establishes *de novo* DNA methylation in mammals. The structure of the Dnmt3a C-terminal domain is similar to the bacterial M. HhaI enzyme, a well-studied prokaryotic DNA methyltransferase. No X-ray structure is available for the complex of Dnmt3a with DNA and the mechanistic details of DNA recognition and catalysis by mammalian Dnmts are not completely understood.

**Results:**

Mutant variants of the catalytic domain of the murine Dnmt3a carrying substitutions of highly conserved N167, R200, and R202 have been generated by site directed mutagenesis and purified. Their methylation activity, DNA binding affinity, ability to flip the target cytosine out of the DNA double helix and covalent complex formation with DNA have been examined. Substitutions of N167 lead to reduced catalytic activity and reduced base flipping. Catalytic activity, base flipping, and covalent conjugate formation were almost completely abolished for the mutant enzymes with substitutions of R200 or R202.

**Conclusions:**

We conclude that R202 plays a similar role in catalysis in Dnmt3a-CD as R232 in M.SssI and R165 in M.HhaI, which could be positioning of the cytosine for nucleophilic attack by a conserved Cys. R200 of Dnmt3a-CD is important in both catalysis and cytosine flipping. Both conserved R200 and R202 are involved in creating and stabilizing of the transient covalent intermediate of the methylation reaction. N167 might contribute to the positioning of the residues from the motif VI, but does not play a direct role in catalysis.

## Background

DNA methylation is a key epigenetic modification in eukaryotes that is essential for various developmental processes, including regulation of gene expression and cell differentiation, genomic imprinting, X-chromosome inactivation, and epigenetic inheritance [1–4]. In mammals, DNA methylation is mediated and maintained by DNA methyltransferases (MTases). These enzymes transfer the methyl group from the cofactor S-adenosyl-L-methionine (AdoMet) to the 5-position of cytosine mainly within CpG dinucleotides [5, 6]. Mammalian genomic DNA methylation is established *de novo* by DNA methyltransferases Dnmt3a and Dnmt3b [6–8]. Pre-existing DNA methylation patterns are primarily maintained by Dnmt1 [8]. However, the roles of the Dnmts are also overlapping, and Dnmt1 contributes to de novo methylation as well as Dnmt3 enzymes are needed for the long term maintainance of DNA methylation [9]. Dnmt3-like protein (Dnmt3L) is a paralogue of the Dnmt3 enzymes that lacks catalytic activity and functions as regulatory protein of the Dnmt3 enzymes [10].

Defects in genes of the *dnmt* family lead to various developmental defects and cancers [2, 11]. Recent cancer genome sequencing revealed *dnmt3a* as one of the most frequently mutated genes across a range of haematological malignancies [12]. These findings emphasize the importance of DNA methylation not only in embryonic development, but also in the maintenance of normal physiology.

Mammalian MTases consist of an N-terminal part, which serves regulatory and targeting functions, and a C-terminal catalytic domain, which contains 10 characteristic amino acid motifs that are conserved among all cytosine-C5 MTases [13, 14]. Unlike Dnmt1, the isolated C-terminal domains of Dnmt3a and Dnmt3b are catalytically active [15]. The C-terminal domains of Dnmt3a and Dnmt3L form a tetrameric complex with two active sites that are separated by about one DNA helical turn [16]. Recent structural studies of Dnmt3a–Dnmt3L-histone H3 complexes revealed that Dnmt3a exists in an autoinhibitory form, where the ADD domain of Dnmt3a interacts with and inhibits the enzymatic activity of the catalytic domain [17]. Histone H3 disrupts this interaction, and thus releases the autoinhibition of Dnmt3a.

Bacterial C5-MTases perform the methylation reaction according to a common mechanism that involves DNA recognition and binding, target cytosine flipping out of the double helix, attack of the conserved cysteine at C6 position and covalent intermediate formation, followed by methyl group transfer from the donor AdoMet, resolution of the intermediate and release of the products [6, 18]. The structure of the Dnmt3a C-terminal domain is similar to the bacterial M. HhaI enzyme, a well-studied prokaryotic MTase [16]. In the Dnmt3a-Dnmt3L structure docked to a DNA molecule with a flipped target cytosine (Cyt), the target Cyt is located between the nucleophile C706 and AdoMet, as in M.HhaI [19, 20]. Both Dnmt3a and Dnmt1 are able to form transient covalent intermediates with DNA containing MTase-trapping base 2-pyrimidinone [21, 22]. Taken together, these observations support the evidence that the same mechanism of C5-methylation is operational in mammalian and bacterial MTases.

Although a few structures of Dnmt3a and its individual domains have been published to date, there are no known structures for the Dnmt3a bound to DNA. Much progress has been made in recent years in our understanding of how MTases function and what the roles are for key amino acid residues in the catalytic process. Mutational analysis of two CpG MTases provided an insight into their catalytic mechanism. The computational model of the prokaryotic CpG MTase M.SssI and its mutational analysis confirmed previously suggested roles for amino acid residues involved in DNA base flipping (S145, R232, T313) and catalysis (E286), and identified a new essential catalytic residue (R230) [23]. Initial mutational analysis of 14 amino acid residues located within ten conserved amino acid sequence motifs has been performed on Dnmt3a-CD in the absence of structure for Dnmt3a-DNA complex [24].

**Fig. 1:**
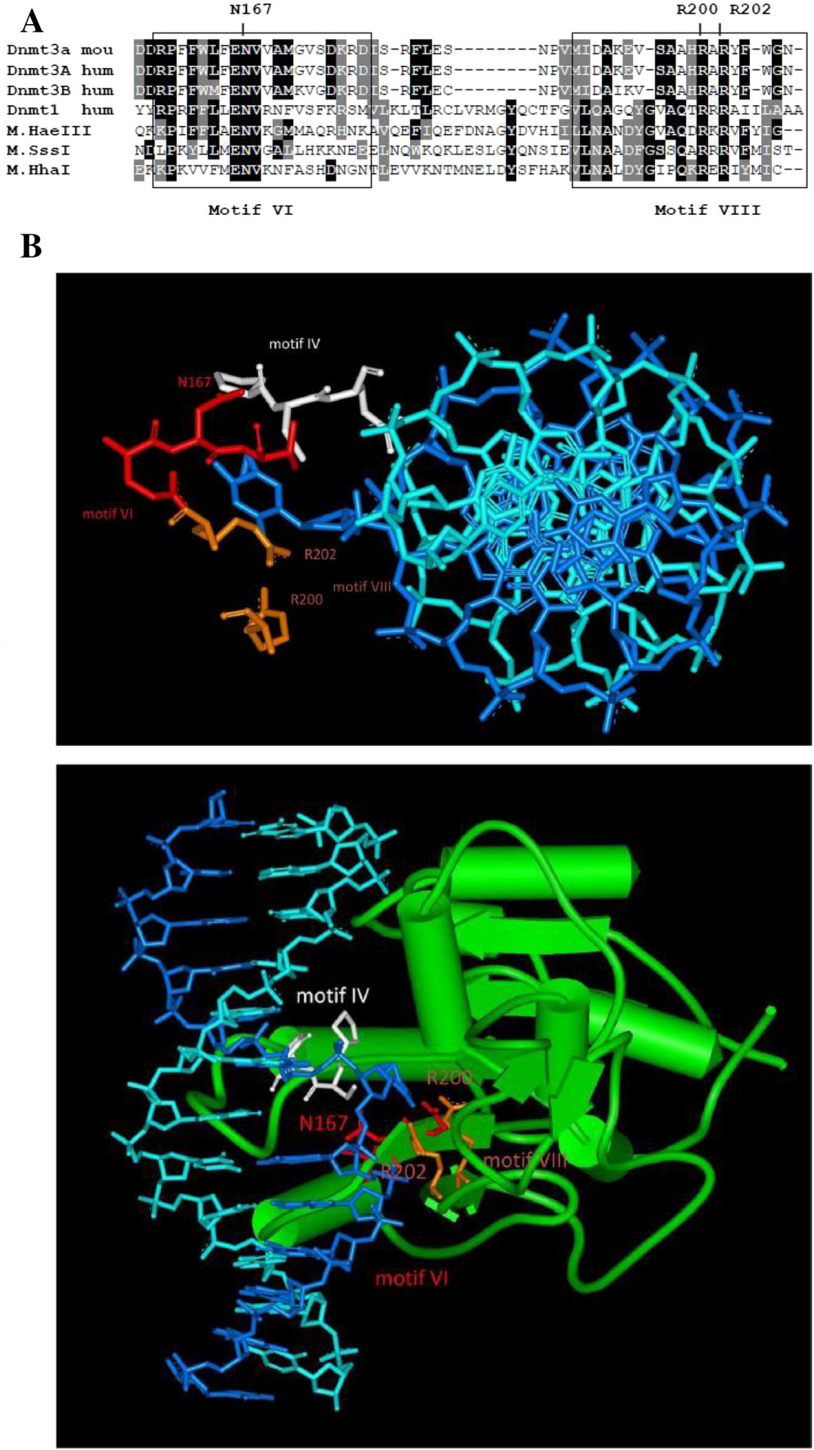
The amino acid residues subjected to mutational analysis. **a** Multiple sequence alignment of mammalian Dnmt3a (from mouse and human), Dnmt3b and Dnmt1 with prokaryotic M.SssI, M.HpaI and M.HhaI. Sequences were aligned by ClustalW algorithm and shaded using BoxShade server. **b** Model of Dnmt3a-CD (green) with short DNA molecule (blue) adopted by superposition of the M.HhaI-DNA complex structure [20] onto Dnmt3a-CD/Dnmt3L tetramer structure [16]. Top (top panel) and side (bottom panel) views. The motifs IV (PCN), VI (ENV) and VIII (QxRxR) are shown

This work is a continuation of the Dnmt3a mutational analysis. We examined in detail the role of N167 (Fig. 1a) of Dnmt3a-CD (motif IV) that might be involved in positioning of the residues from motif VI [24] (the corresponding numbers of the amino acids in full length Dnmt3a are indicated in Table 2). R200 and R202 of Dnmt3a-CD (motif VIII, Fig. 1a) were selected on the basis of our computational model of the ternary M.SssI•DNA•AdoHcy complex and results of mutational analyses of Dnmt3a-CD, M.SssI and M.HhaI MTases [23–25]. The mutated amino acid residues are shown on the model of Dntm3a/DNA complex (Fig. 1b). These residues may play a role in catalysis of cytosine-C5 methyltransferases. Mutant forms of Dnmt3a-CD were generated by site-directed mutagenesis and key steps of the methylation reaction were studied biochemically.

## Methods

### Chemicals, oligodeoxynucleotides and enzymes

[CH_3_-^3^H]AdoMet (15 Ci/mmol, 66.7 μM) was purchased from Amersham Biosciences (Little Chalf-ont, U.K.). AdoHcy was from Sigma (St. Louis, MO). All unmodified or 2-aminopurine-containing oligodeoxynucleotides were purchased from Syntol (Moscow, Russsia). The oligodeoxynucleotide containing 2-pyrimidinone was synthesized by S.N. Mikhailov (Moscow, Russia), and its composition was confirmed by mass spectrometry using a Bruker Daltonics Reflex II MALDITOF MS instrument (Bruker, Billerica, MA). The fluorescent label (FAM) was 5′-linked to oligonucleotides by a hexamethylene linker. Oligodeoxynucleotide sequences are presented in Table 1.

**Table 1:** Oligodeoxynucleotide sequences^*a*^

Designation	Sequence
GCGC	5'-CTGAATACTACTTGCGCTCTCTAACCTGAT-3'
fGCGC	5'-FAM-CTGAATACTACTTGCGCTCTCTAACCTGAT-3'
GBGC	5'-CTGAATACTACTTGBGCTCTCTAACCTGAT-3
CGCG	3'-GACTTATGATGAACGCGAGAGATTGGACTA-5'
CGMG	3'-GACTTATGATGAACGMGAGAGATTGGACTA-5'
fCG	5'- FAM-GAGCCAAGCGCACTCTGA
GP	3'-CTCGGTTCGPGTGAGACT-5'

^*a*^M, 5-methylcytosine; В, 2-aminopurine; P, 2-pyrimidinone; FAM (f), 6-carboxyfluorescein

Oligodeoxynucleotide duplexes were prepared by annealing the complementary strands in 10 mM Tris–HCl (pH 7.9) buffer containing 50 mM NaCl.

### Plasmids and mutant enzymes

Expression constructs of Dnmt3a-CD and Dnmt3L have been described previously [16, 24]. Selected amino acids of CD-Dnmt3a were mutated using a PCR-megaprimer mutagenesis method as described [24]. Mutagenesis was confirmed by Sanger sequencing. Dnmt3a-CD and Dnmt3L were overexpressed in BL21(DE3) *E.coli* as N-terminally His_6_-tagged or His_8_-tagged proteins, respectively, and purified according to the previously described protocol [26]. Mutant forms of Dnmt3a-CD were isolated and purified in the same way as the wild type (wt)-enzyme. The concentrations of protein samples were measured by standard Bradford technique.

### DNA methylation

DNA methylation was monitored by the time dependencies of amount of tritium-labeled methyl groups transferred from [CH_3_-^3^H]AdoMet to cytosine by the enzymes as described [26]. The reaction mixtures contained 1.5 μM G**CG**C/C**GC**G, 2 μM [CH_3_-^3^H]AdoMet and 0.89 μM Dnmt3a-CD or its mutant form in Dnmt3a reaction buffer (10 mM Tris–HCl, pH 7.9, 50 mM NaCl, 2 mM mercaptoethanol). Aliquots of the reaction mixtures were pipetted onto DE-81 paper disks (Whatman) after 10–40 min intervals. Amount of incorporated radioactivity was counted and plotted using Origin 6.0 software (OriginLab).

### DNA binding

DNA binding was monitored by fluorescence polarization. Fluorescence polarization (P) was measured at 25 °C in quartz cuvette «Hellma» with a 1-cm pathlength using a Cary Eclipse (Varian) spectrofluorimeter, with excitation at 495 nm and emission at 520 nm, and slit widths were 10 nm. 10 nM fG**CG**C/C**GC**G was preincubated with 0.1 mM AdoHcy in Dnmt3a reaction buffer, and fluorescence polarization of free DNA (P_0_) was measured. Then Dnmt3a-CD or its mutant variant was added as 1–5 μL aliquots of the stock solution (2.91 μM) up to a final concentration of 600–800 nM, and the polarization value (P) recorded at 1 min after each addition was measured. Titration curves were analyzed using SCIENTIST software from MacroMath (St.Louis, MO). Dissociation constants, *K*_d1_ and *K*_d2_ and the product *K*_d1_*K*_d2_ were obtained from the model of cooperative binding as described in [27].

### Base flipping

Base flipping was studied by fluorescence spectroscopy as described in [23]. Dnmt3a-CD (300 nM) or its mutant was incubated with Dnmt3L (300 nM) at 37 °C in Dnmt3a reaction buffer containing 0.1 mM AdoMet and 200 nM 2-aminopurine-containing duplex G**BG**C/C**GM**G. The spectra were recorded 10 min following incubation. Background correction was implemented by subtraction of the control spectrum obtained with a reaction mixture containing G**CG**C/C**GM**G instead of 2-aminopurine substituted DNA. The fluorescence enhancement was calculated for the individual mutant relative to the wt enzyme.

### Detection of MTase-DNA conjugates

Reactions contained 200 nM FAM-labeled duplex f**CG**/**GP**, 0.1 mM AdoHcy and 2 μM Dnmt3a-CD (wt or mutant) in Dnmt3a reaction buffer. After incubation at 4 °C for 60 min the samples were incubated with 1 % SDS at room temperature for 10 min. Samples were subjected to electrophoresis in 12.5 % denaturing Laemmli polyacrylamide gels containing 0.1 % SDS. The gels were analyzed by fluorography using FUJIFILM FLA-3000 (Japan).

## Results

**Fig. 2:**
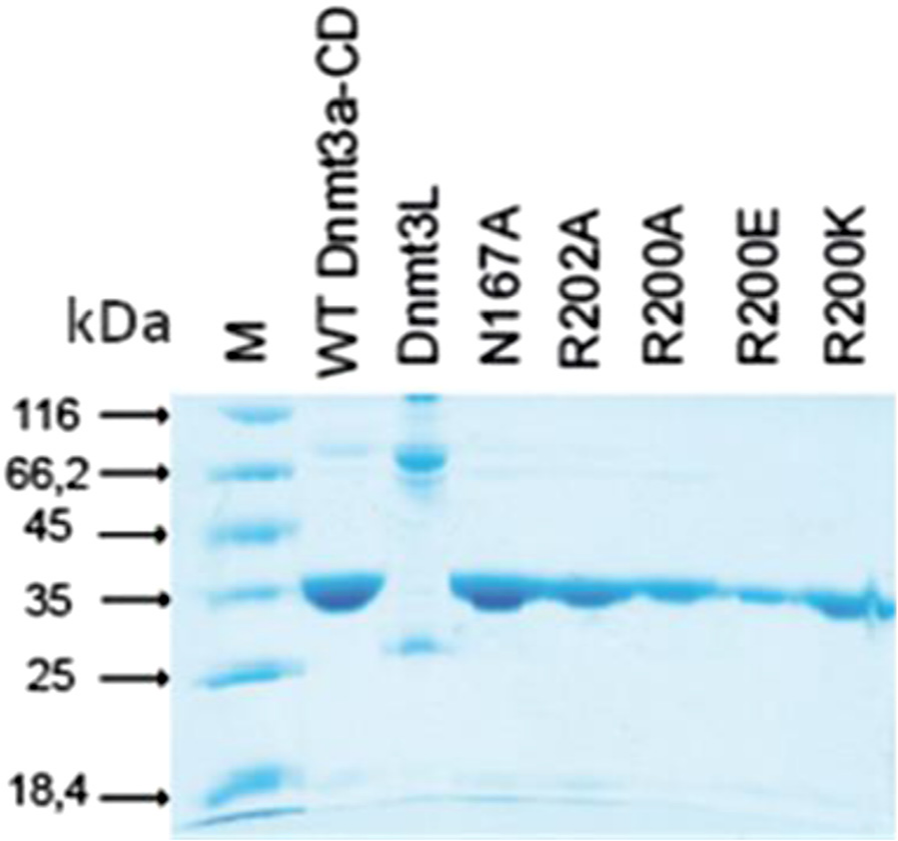
Coomassie blue-stained SDS-polyacrylamide gel showing the purified recombinant Dnmt3a-CD protein, its variants and Dnmt-3 L

Amino acid exchanges of the conserved residues N167, R200 and R202 of the murine Dnmt3a-CD (Fig. 1) were introduced by site-directed mutagenesis. The Dnmt3a-CD mutants were expressed in *E.coli* as C-terminal His_6_-proteins and purified in soluble form (Fig. 2).

### DNA methylation activity

**Fig. 3:**
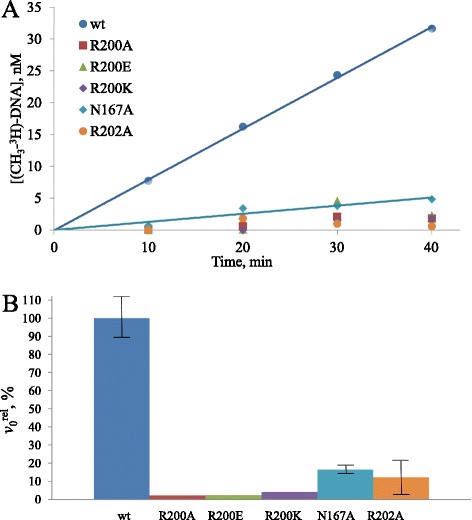
Time courses of methylation of oligodeoxynucleotide duplex by wt or Dnmt3a-CD variants. **a** The reaction mixtures contained 1.5 μM G**C** **G**C/C**G** **C**G, 0.89 μM Dnmt3a-CD or its variants, and 2 μM [CH_3_-^3^H]AdoMet. **b** Relative initial methylation rates (*v*_0_^rel^,%) for wt and Dnmt3a-CD variants. The error bars represent the SDs derived from at least three independent experiments

DNA methylation activity of the purified enzymes was quantitatively assessed by calculating the initial rate of methylation (*v*_0_). Time dependencies were determined for the incorporation of radioactive labeled methyl groups from S-[methyl-^3^H]-AdoMet to unmethylated 30-mer DNA duplex G**CG**C/C**GC**G, which harbors one centrally placed CG site (in bold, methylated cytosine is underlined) by the wild type (wt) Dnmt3a-CD or its mutants. The reactions were carried out under conditions where the DNA concentration was 1.7-fold greater than that of the enzyme. Dnmt3a-Dnmt3L heterodimers might have higher intrinsic activity than Dnmt3a-Dnmt3a homooligomers, in part because Dnmt3L favors the catalytically competent closed conformation of the Dnmt3a active-site loop [16, 19]. However, functional analysis of mutants can be performed in the absence of Dnmt3L since Dnmt3a-CD oligomers are catalytically active enough to achieve significant signal in the methylation assay. The time courses for all mutants were linear, as it is exemplified for the N167A mutant (Fig. 3a). The obtained relative methylation rates are presented in Fig. 3b and Table 2. The Ala, Glu and Lys substitutions for R200 led to an almost complete loss of methylation activity. This result suggests that R200 is extremely important for Dnmt3a-CD functioning. The N167A and R202A also displayed significantly reduced DNA methylation rates (6- and 8-fold, respectively) that is in accordance with previous findings [24].

**Table 2:** Methylation activity, DNA binding affinity, and 2-aminopurine base flipping by Dnmt3a-CD mutants^a^

Exchange	Corresponding residue in full length Dnmt3a	*K*_d1_, nM	*K*_d2_, nM	*K*_d1_ *K*_d2_, nM^2^	Relative binding affinity	*v*_0_, nM/min	*v*_0_^rel^, %	Relative intensity of 2-aminopurine fluorescence, at 370 nm, %
wt		73 ± 17	14 ± 5	1022	1.00	0.83 ± 0.09	100	100
N167A	N753	n.d.	n.d.	n.d.	0.7^b^	0.14 ± 0.02	17	29.4
R200A	R786	79 ± 17	14 ± 6	1106	0.92	<0.020	2	3.9
R200E	R786	159 ± 37	33 ± 12	5247	0.19	<0.022	3	5.3
R200K	R786	55 ± 14	9 ± 3	495	2.06	<0.035	4	15.4
R202A	R788	n.d.	n.d.	n.d.	1.20^b^	<0.100	12	0.9

^a^The amino acid numbers in Dnmt3a catalytic domain are given relative to the murine full-length Dnmt3a (NCBI reference sequence NP_001258682.1). n.d. designates not determined

The given values are representative of two or more experiments

^b^Data from ref. [24]

### DNA binding affinity

**Fig. 4:**
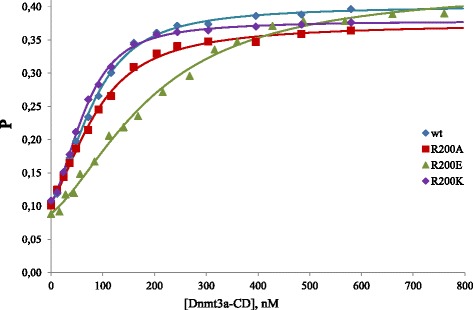
Binding curves obtained by titration of the fluorescein labeled unmethylated oligodeoxynucleotide duplex fG**C** **G**C/C**G** **C**G (10 nM) with increasing amounts of Dnmt3a-CD or its variants in the presence of AdoHcy (0.1 mM). *P* represents the polarization value. The lines signify the best fits to the model described in [27]. Enzyme concentration is shown in terms of monomer

Binding to DNA is the first step of the methylation reaction. Changes in the methylation rates of mutant enzymes may result from their altered substrate binding affinities. Complex formation was monitored by changes in fluorescence polarization using a FAM-labeled oligodeoxynucleotide duplex fG**CG**C/C**GC**G binding substrate. Binding measurements were performed in the presence of the cofactor product, AdoHcy. Binding curves displayed cooperative binding (Fig. 4). The data were analyzed according to the previously published model that includes binding of two Dnmt3a-CD complexes to 30-mer DNA duplexes to form a catalytically more competent multimeric complex [26, 27]. In this model, individual binding constants for binding of the first Dnmt3a complex (K_d1_) and the second Dnmt3a complex (K_d2_) were obtained. The product K_d1_K_d2_ was used as a binding characteristic for the mutant enzymes (Table 2). All R200 substitutions did not lead to a loss of binding cooperativity. Replacement of R200 to Ala or Lys did not affect DNA binding significantly. However, there was a 5-fold decrease in *K*_d1_*K*_d2_ for the R200E variant. This indicates that R200 does not play crucial role in DNA binding, but the reversal in polarity of this residue led to the deterioration of binding because of possible repulsion of negative charges of glutamate and DNA phosphate backbone. As it was shown previously, the N167A variant displayed slightly decreased binding affinity, whereas binding of R202A to DNA was not affected (Table 2 and [24]). Defects in methylation activity for N167A and R202A (Table 2) cannot be explained by their slightly reduced DNA binding, therefore, it was necessary to investigate next steps of the methylation reaction.

### Base flipping

**Fig. 5:**
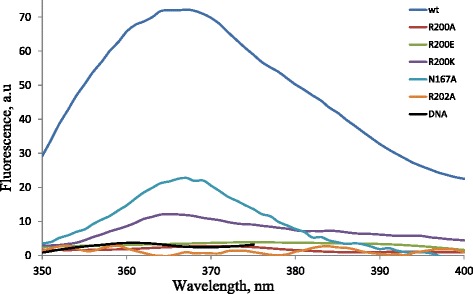
Base flipping induced by Dnmt3a-CD or its variants in complex with Dnmt3L. Emission spectra were recorded for the complex of Dnmt3a-CD (300 nM) with Dnmt3L (300 nM) and 2-aminopurine containing hemimethylated DNA duplex G**B** **G**C/C**GM**G (200 nM) in the presence of AdoMet (0.1 mM)

Flipping of the target cytosine by Dnmt3a-CD and its mutants in the presence of Dnmt3L was analyzed using hemimethylated oligonucleotide duplex G**BG**C/C**GM**G containing the fluorescent base 2-aminopurine in place of the target cytosine. The fluorescence of 2-aminopurine is highly quenched in the helix due to stacking interactions with neighboring bases, and increases strongly when the base is flipped out [28, 29]. Dnmt3a-CD alone, when mixed with 2-aminopurine containing DNA, produced low signal with high signal to noise ratio (data not shown). To increase the enzymatic activity of the complex, Dnmt3L was added. The changes in the 2-aminopurine fluorescence upon mixing of the substrate with enzymes are given in Table 2, examples of the emission fluorescence spectra are shown in Fig. 5. The complex of wt Dnmt3a-CD-Dnmt3L with duplex G**BG**C/C**GM**G gave a strong fluorescence signal with a characteristic maximum at 365–370 nm in the presence of the cofactor AdoMet (Fig. 5). The fluorescence signal observed for N167A mutant was 3.5-fold lower than that for the wt-enzyme. R200K mutant, despite its elevated binding affinity (Table 2), showed 7-fold reduction in the fluorescence signal. Other mutants (R200A, R200E, R200A and R202A) produced much lower signal (Table 2 and Fig. 5), which is consistent with the loss of base flipping.

### Covalent intermediate formation

One of the key steps of the DNA methylation reaction is the formation of the transient covalent complex between the enzyme and its substrate DNA. This step was studied for wt Dnmt3a and its mutant variants using an oligonucleotide duplex f**CG**/**GP** containing 2-pyrimidinone at the place of the target cytosine and terminal fluorescent label for fluorographic detection. Normally, the nucleophylic attack of the highly conserved cysteine of the motif IV at the C6 position of the cytosine ring produces the covalent conjugate, which triggers the subsequent methyl group transfer from AdoMet to the C5 carbon atom. The covalent complex is resolved by deprotonation at C5 leading to ß-elimination of the cysteine residue [18]. Conjugates of C5-MTases with DNA containing 2-pyrimidinone instead of the target cytosine are stable due to the retardation of β-elimination and can be detected by gel electrophoresis [23, 30].

**Fig. 6:**
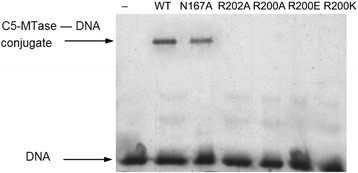
Analysis of covalent complex formation of Dnmt3a-CD wt and its variants with 2-pyrimidinone-substituted DNA duplex f**C** **G**/**G** **P**. Fluorograph of a 12.5 % denaturing Laemmli polyacrylamide gel. The samples were treated with 1 % SDS for 10 min at room temperature

The wt Dnmt3a-CD forms SDS-resistant complexes in the presence of AdoHcy (Fig. 6). Covalent intermediate was detected with the N167A mutant enzyme as well, although with slightly reduced efficiency, which reflects its reduced binding affinity (Table 2). No covalent complex was produced in the case of mutant enzymes carrying substitutions of R200 and R202. This fact, together with the abolished base flipping (Fig. 5) demonstrates the outstanding importance of these two Arg residues in the enzyme functioning.

## Discussion

The methylation reaction of the C5-MTases is thought to consist of the following steps [6, 15, 31]. The variable region TRD and motif IX initiate recognition and binding of target the DNA sequence. Motifs I-III constitute the binding site for the methyl group donor AdoMet. The glutamic acid of the motif VI (ENV) transiently protonates the endocyclic N3 nitrogen atom of the pyrimidine ring. That facilitates the nucleophylic attack of the highly conserved cysteine of the motif IV (PCQ) at the C6 position of the cytosine ring and the subsequent transfer of the methyl group from AdoMet to the C5 carbon atom. Simultaneously, the glutamate from the ENV motif stabilizes the flipped base via the contact with the N4 exocyclic amino group and the glutamine from TRD (Q237 in M.HhaI) stabilizes the orphan guanine. The general base involved in the proton abstraction from the transient covalent intermediate is not known. However, theoretical calculation showed that a hydroxide anion rather than basic residue(s) in the active site may be involved in the deprotonation step [32].

Figure 1b shows a model of Dnmt3a-CD with short DNA molecule adopted by superposition of the M.HhaI-DNA complex structure [20] onto Dnmt3a-CD/Dnmt3L tetramer structure [16]. The motif IV (PCN instead of PCQ) contains the catalytic cysteine (C120). The motifs VI (ENV) contains N167 and motif VIII (QxRxR) contain R200 and R202. In this work, we studied the role of three conserved residues N167, R200 and R202 in Dnmt3a in detail as described in the next paragraphs.

### R200

The R200 residue in Dnmt3a-CD is one of the highly conserved amino acids of the motif VIII (Qx**R**xR). It corresponds to R163 in M.HhaI, R230 in M.SssI, and R228 in bacterial CpG-specific MTase M.MpeI [33]. There are some indications for a role of this residue in the cytosine methylation reaction mechanism involving different prokaryotic C5 MTases. First, besides the glutamic acid of the motif VI (ENV), this arginine residue could also play critical roles in the protonation of N3 position in the target cytosine. Computer simulation studies suggested that the function of the R163 in M.HhaI could be to donate a proton via a bridging water molecule to N3 of the target cytosine [34]. Quantum mechanical/molecular mechanics analysis further indicated that the two arginine residues R163 and R165 (equivalent to R200 and R202 in Dnmt3a-CD) in the conserved motif VIII may be essential for stabilizing the delocalized electrons in cytosine moiety of the transient covalent intermediate through their intrinsic electrostatic interactions [31, 32]. A crystal structure of M.MpeI with DNA demonstrated that residues R228 and R230 (equivalent to R202 in Dnmt3a-CD) are close to the flipped base (approximately 3.0 Å) and donate one or two hydrogen bonds to its O2 [33]. Analysis of the structural model of the M.SssI•DNA•AdoHcy complex revealed that R230 might be involved in maintaining the proper orientation of the target cytosine. In M.SssI, alanine substitution of R230 did not affect DNA binding of the enzyme, whereas its methylation activity was almost completely abolished, and the efficiency of conjugate formation of this mutant with 2-pyrimidinone containing DNA was greatly reduced compared to the wt enzyme [23].

In Dnmt3a, the exchange of R200 to Ala, Glu or Lys led to a complete loss of methylation activity and abolished covalent intermediate formation ability. The target cytosine flipping in the presence of Dnmt3L was undetectable in the case of R200A and R200E, while R200K mutant was able to flip out the cytosine, albeit with only 16 % efficiency compared to the wt-enzyme. Regarding the substrate binding, the most appreciable effect was detected when R200 was substituted for Glu. The product *K*_d1_*K*_d2_ was 5-times higher than that of the wt-Dnmt3a indicating a weakening of binding. This can be explained by a repulsion of a negatively charged carboxyl group of the glutamic acid side-chain and the phosphate groups of the DNA backbone. The other R200 substitutions had no effect (R200A) or slightly improved substrate binding (R200K). Taken together, the effects of arginine to alanine substitution in Dnmt3a were more considerable than that of M.SssI. The lack of catalytic activity displayed by the R200A mutant is consistent with the high degree of conservation of this arginine in C5-MTases [35] and its extreme importance in target cytosine positioning, i.e. in both catalysis and cytosine flipping.

### R202

The other highly conserved Arg in motif VIII (QxRx**R**) is R202 in Dnmt3a-CD. According to the X-ray data, the side chains of the corresponding R165 (in M.HhaI) [20] and R228 (in M.MpeI) [33] form a direct hydrogen bond with the O2 of the target cytosine ring. Homology modeling/molecular dynamic stimulation study predicted the same hydrogen bonding for the equivalent R162 of M.HgiDII [36]. It was theoretically proposed that this Arg residue should stabilize the intermediate with delocalized electrons in the cytosine ring by forming a salt-bridge with O2 [37]. Functional and structural studies of the R165A mutant of M.HhaI showed that this Arg is important for base flipping, as well as for positioning the cytosine for the nucleophilic attack by the conserved C81 [25].

The R202A mutant of the murine Dnmt3a-CD showed wt level of DNA binding affinity and a very low catalytic activity [24]. Similarly, in M.HhaI, an Ala replacement of R165 led to a fourfold increase in DNA binding affinity and a 10^5^-fold decrease in catalytic rate [25]. The loss of 2AP fluorescence for the complex of R202A with 2AP containing DNA (Fig. 5 and Table 2) and the lack of the transient covalent intermediate (Fig. 6) were observed. These effects are similar to R232A of M.SssI, where there were no significant changes in DNA binding, but strong reduction in methylation rate and intensity of 2-aminopurine fluorescence were observed [23], suggesting that R202 plays a similar role in catalysis in Dnmt3a as R232 in M.SssI and R165 in M.HhaI. This Arg residue could participate in positioning of the flipped target base and creating and stabilization a reactive enamine functionality that could attack the methyl group of AdoMet.

### N167

In M.HhaI, motif IV and VI are connected by a highly conserved H-bond between N120 (ENV) and the carbonyl group of P80 (PCQ) that contributes to the positioning of the two catalytic motifs with respect to each other. Mutational analysis of the corresponding asparagine residue (N167) in Dnmt3a was performed in [24] to test for the function of this contact. It was shown that N167A mutant retains normal AdoMet and DNA binding, but a 10-fold decrease in methylation rate was observed. It was concluded that alterations at any residue in motif VI interfere with the accurate positioning of the glutamate residue, which is directly involved in catalysis.

Fluorescent spectroscopy assays revealed that the Dnmt3a N167A mutant retains an ability to flip target cytosine out of DNA with 32 % efficiency compared to the wt-enzyme (Fig. 5 and Table 2). A detectable quantity of covalent adducts with 2-pirimidinone-containing DNA was observed (Fig. 6). Since the effects were not so strong comparing to R200 and R202 substitutions, we conclude that N167 might contribute to the positioning of residues from motif VI, but does not play a direct role in catalysis.

## Conclusion

In summary, our data demonstrate the key role of two highly conserved arginine residues from the motif VIII, R200 and R202, in catalysis of the cytosine methylation by Dnmt3a-CD. They are involved in positioning of the flipped target base and together with E166 from motif VI and C120 from the catalytic loop (motif IV) in creating and stabilization a reactive transient covalent intermediate that could attack the methyl group of AdoMet. This characteristic seems common for the closely related motif VIII arginines of all bacterial and mammalian cytosine C5 MTases. The information obtained may be also relevant for the explanation of the consequences of the multiple mutations in the gene encoding DNMT3A recently reported in patients with various haematological malignancies [12].
